# Ewing sarcoma of the mandible mimicking an odontogenic abscess – a case report

**DOI:** 10.1186/1746-160X-4-24

**Published:** 2008-11-05

**Authors:** Martin Gosau, Daniel Baumhoer, Stefan Ihrler, Johannes Kleinheinz, Oliver Driemel

**Affiliations:** 1 Department of Cranio-Maxillo-Facial Surgery, University of Regensburg, Germany; 2 Institute of Pathology, University of Basel, Switzerland; 3 Institute of Pathology, University of Munich, Germany; 4 Department of Cranio-Maxillo-Facial Surgery, University of Münster, Germany

## Abstract

Ewing sarcoma (ES) of the mandible is rare and can be mistaken for inflammation of dental origin. We present a 24-year old male patient which underwent radical tumour surgery and primary reconstruction with a microvascular osteoseptocutaneous free fibular flap as well as postoperative adjuvant chemotherapy. Incomplete osseous tumour resection required a second intervention. This case report recapitulates the clinical and histopathological findings in oral ES, demonstrates its sometimes difficult diagnosis and discusses the (dis-)advantages of primary osseous reconstruction in ablative tumour surgery.

## Introduction

Primary malignant tumours of the jaws are rare and especially the diagnosis and treatment of Ewing sarcoma (ES) (ICD-0 code 9260/3) can be challenging. Less than 3% of all ES originate in the maxillofacial region, usually involving the mandible [1,2]. 90% occur in the first three decades of life and males are more often affected than females (male:female = 3:2) [1,3]. Clinical symptoms such as swelling, pain and sensory disturbances are rather unspecific and can sometimes be misleading.

The following case-report is presented to recapitulate the clinical and histopathological findings in oral ES, demonstrate its sometimes difficult diagnosis and discuss the (dis-) advantages of primary osseous reconstruction in ablative tumour surgery.

## Clinical history

Four weeks before admission a 24-year-old man noticed a swelling in the right floor of the mouth. Assuming that the tumour was an acute dental abscess the attending dentist incised the tumour and prescribed antibiotics. A temporary decline of the symptoms was noted and root channel treatment, root amputation and splinting of the teeth 43 – 45 due to negative sensibility and loosening was performed. However, the pathologic process of the right mandible enlarged constantly and the patient was referred to our department three weeks after initial treatment.

**Figure 1:**
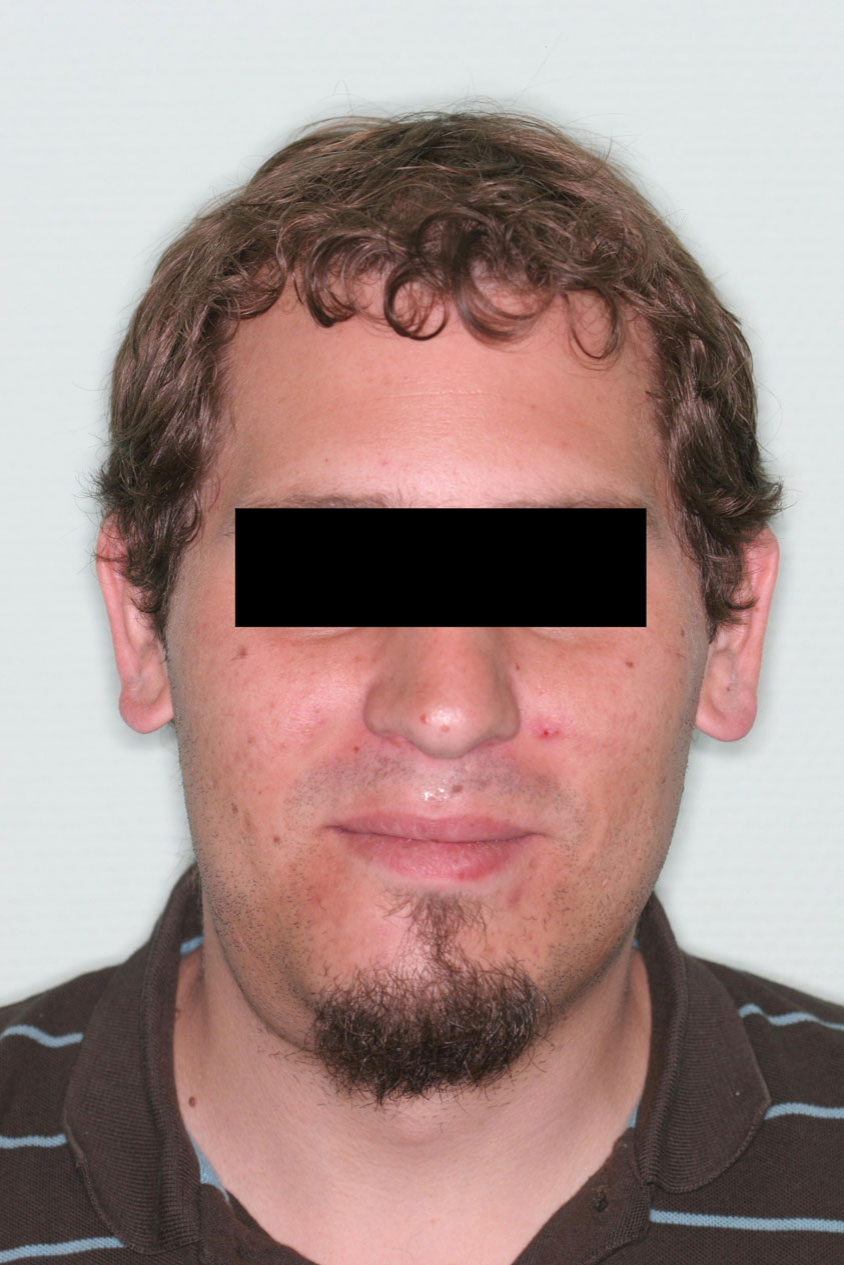
Photograph at initial examination showing a submanibular swelling on the right side.

**Figure 2:**
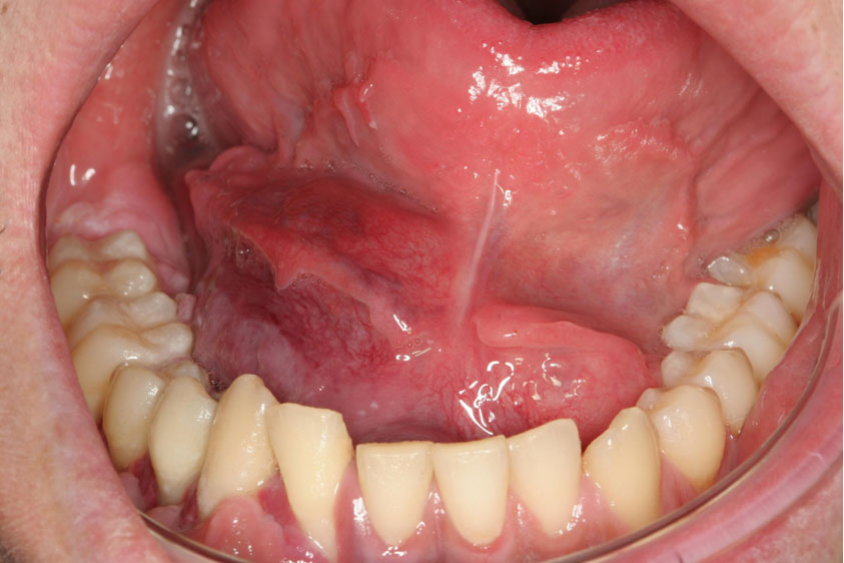
Intraoral photograph taken at initial examination showing the tumour mass coming from the mandible and infiltrating the gingiva as well as the floor of the mouth.

**Figure 3:**
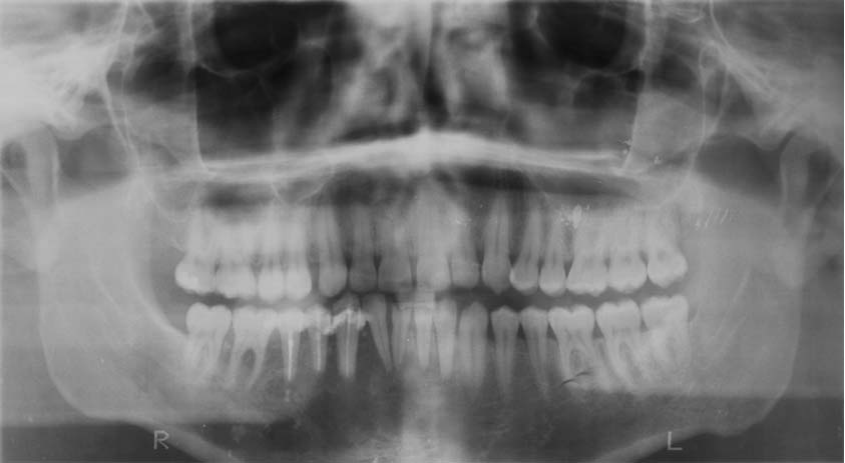
Panoramic radiograph, taken two weeks before presentation by the attending dentist, showing an osteolytic process in the right mandible and the teeth 43–45 after root channel treatment and root amputation.

**Figure 4:**
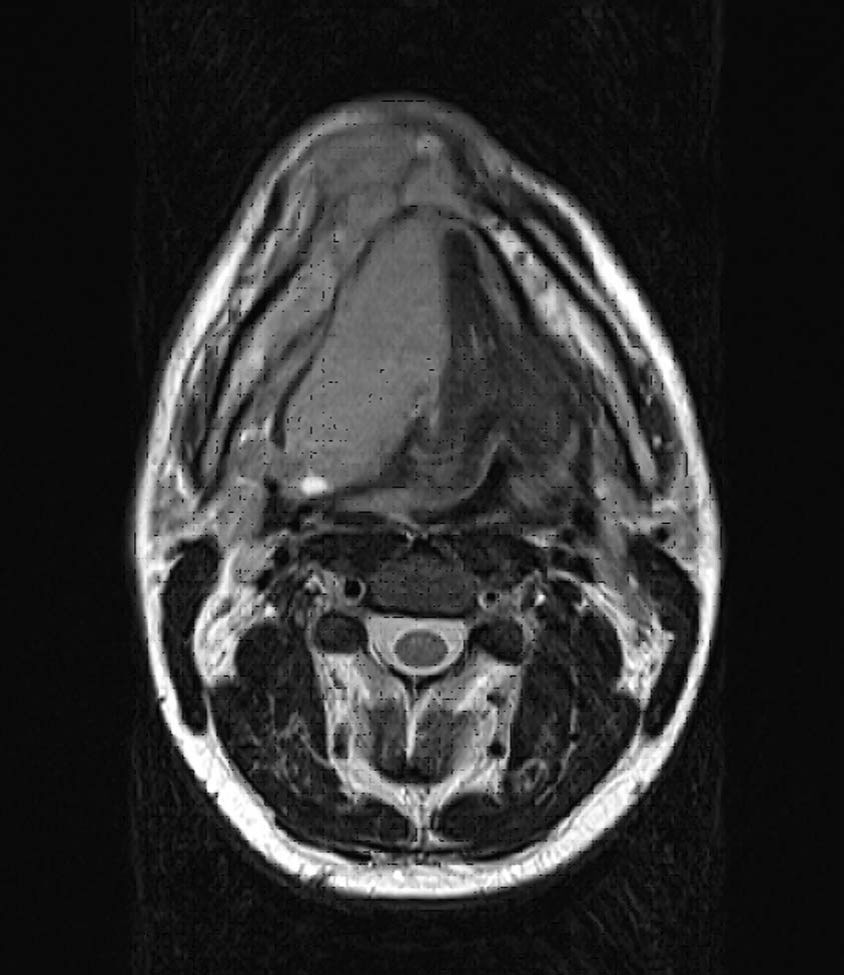
Axial MRI scan revealing a bone destroying mass of approximately 7 × 8 × 6 cm^3 ^surrounding the mandible and massively infiltrating the soft tissue of the floor of the mouth and the tongue.

**Figure 5:**
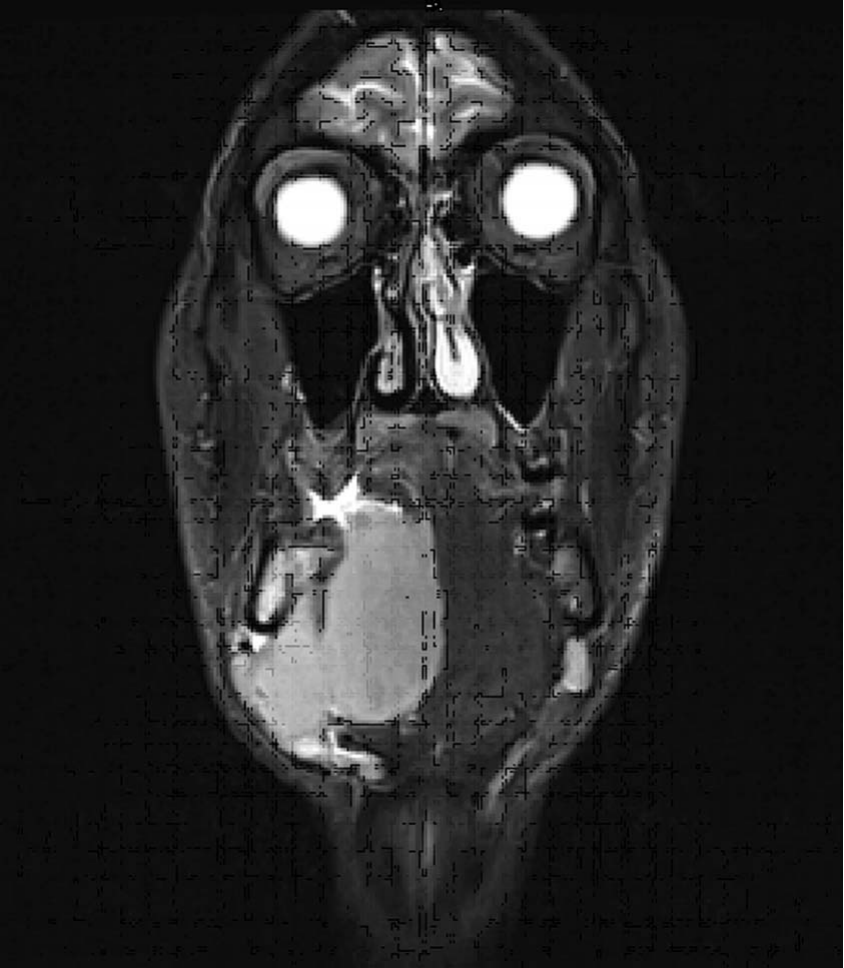
Coronal MRI scan revealing a bone destroying mass of approximately 7 × 8 × 6 cm^3 ^surrounding the mandible and massively infiltrating the soft tissue of the floor of the mouth and the tongue.

**Figure 6:**
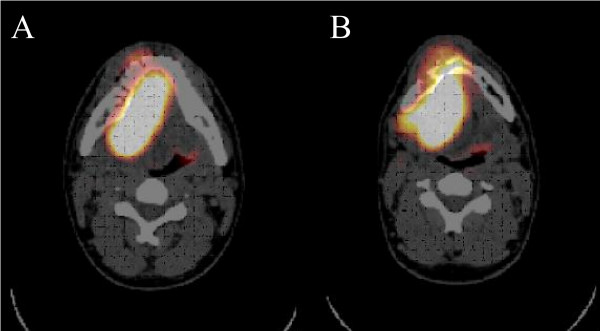
**a, b: Radionuclid (FDG) enhancement in the right floor of the mouth expanding over the midline. ** No further enhancement in the whole body scan visible.

On admission the patient was in good general condition. Body temperature was normal, blood examination showed elevated CRP (7.92 mg/l) and regular leucocyte count (8.71/nl). Physical examination revealed an asymmetric swelling of the right mandible with inconspicuous overlaying mucosa and skin (fig. 1, 2). The tumour was solid, slightly painful, well-circumscribed and measured 7 cm in diameter. Hypaesthesia was evident in the right lower lip. The teeth 32–46 were loosened and showed negative sensibility. A panoramic radiograph taken two weeks before by the attending dentist showed a diffuse radiolucency with ill-defined borders located in the right mandibular corpus (fig. 3). Additional CT-, MRI- and PET-scans confirmed the osteolytic mass with extensive soft tissue infiltration but without evidence of metastatic disease (fig. 4, 5, 6).

**Figure 7:**
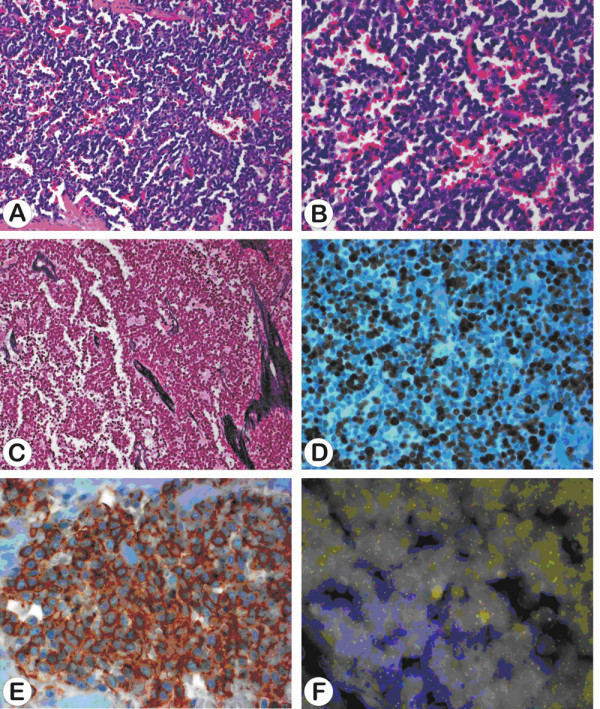
**a-f: Sheets of uniform and densely packed small cells showing round nuclei with finely granular nuclear chromatin (Haematoxylin and Eosin, a 200×, b 400×) and lack of reticulin fibres (Novotny reticulin stain, c 400×).** The proliferation index is high (approximately 80% of cells showing immunoreactivity against MIB1, d 400×) and tumour cells demonstrate strong positivity for CD99 (e 630×). FISH analysis using an EWSR1(22q12) dual colour break apart rearrangement probe demonstrates tumour cells with separate orange and green signals indicating t(11;22)(q24;q12) (f 630×).

Incisional biopsy identified ES (grading according to FNCLCC: grade 3; diagnosis confirmed by Prof. Leuschner, sarcoma reference centre, Kiel and Prof. Jundt, bone tumour reference centre, Basel). Histologically, the tumour was composed of uniform small round cells with indistinct cytoplasm and round nuclei with finely dispersed chromatin (fig. 7a, b). Lack of reticulin fibres (fig. 7c), high proliferations index (fig. 7d) and strong immunoreactivity against CD99 (fig. 7e) underlined the diagnosis of ES. Finally, the characteristic translocation (11;22)(q24;q12) was detected using Fluorescence in situ Hybridisation (fig. 7f).

**Figure 8:**
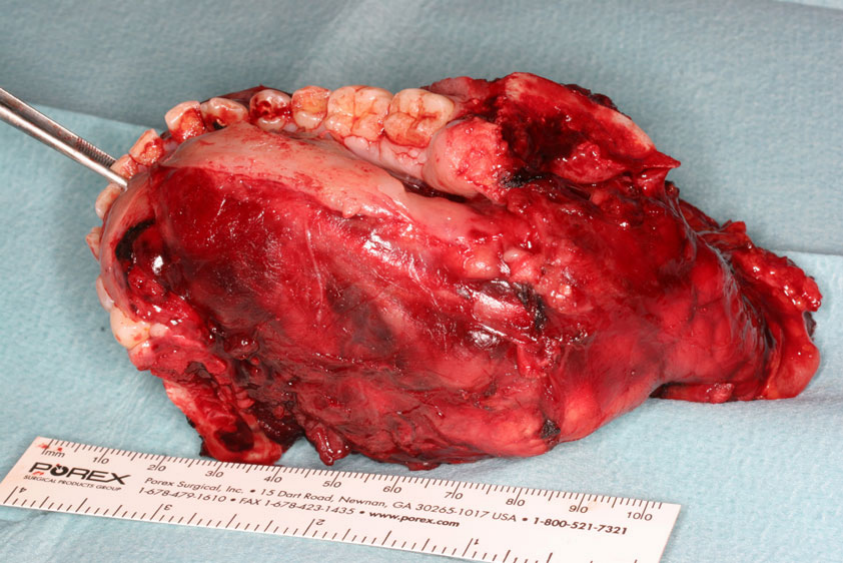
Mandibular resection specimen.

The patient underwent radical tumour surgery with subtotal mandibulectomy and cervical lymph node dissection (Fig. 8). Reconstruction was performed using a microvascular osteoseptocutaneous fibular free flap. Tumour free soft tissue margins were confirmed intraoperatively. Histopathological examination of the resected bone, however, showed infiltration of both mandibular resection margins necessitating re-excision. The bony defects were filled with free iliac crest grafts. Two weeks postoperatively the patient underwent chemotherapy according to a standardized study protocol (CWS-2002P-study).

## Discussion

Swelling, pain, increased CRP, leukocytosis, and elevated temperature may be the first signs of oral ES, occurring also in odontogenic infections [4-6]. In the present case additional apical osteolysis, loss of sensibility and loosening of teeth resulted in root channel treatment, root amputation and splinting of the teeth 43 – 45 with delay of the diagnosis. Root amputation surgery due to large apical osteolytic lesions therefore always requires histopathological evaluation [7].

ES is a poorly differentiated neuroectodermal tumour with small, round and blue cells [5,6]. More than 90% of cases show a characteristic translocation t(11;22)(q24;q12) resulting in the fusion of the EWS and FLI-1 genes (Fig. 7f). This gene rearrangement causes a fusion product which functions as an oncogenic aberrant transcription factor with structural variability and potentially prognostic impact [8]. Immunoreactivity against FLI-1 and CD 99 can help to confirm the diagnosis [6].

Treatment of ES should include wide surgical resection and (neo-)adjuvant chemotherapy [6].

In the presented case a resection with intraoperative histological control of the soft tissue margins and primary reconstruction of the mandible with a microvascular fibular free flap was performed. On the one hand, immediate bony and soft tissue reconstruction plays a key role in minimizing the deformity created by tumour resection and preventing wound contraction and displacement of bony segments. The combination of ablative and reconstructive surgery within a single procedure reduces the overall treatment time and provides the best aesthetic and functional results [9,10]. On the other hand it is not possible to control bone margins intraoperatively. Although wide resection based on MRI and CT scans were planned, residual tumour infiltrates were detected in both bony resections margins. Even if MRI scans are performed to assess intramedullar tumour extension, microscopic tumour infiltrates are impossible to detect radiologically [11]. This raises the question whether the aesthetic and functional favourable primary reconstruction or the secondary reconstruction with better control of the bony tumour margins should be favoured.

## Conclusion

Oral Ewing sarcoma is a rare tumour entity which can mimic odontogenic inflammation. If root amputation surgery is performed due to an extensive radiolucent lesion, histopathological evaluation should be mandatory. Primary bony reconstruction bears the risk of second intervention when dealing with ES in the mandible.

## Consent

Written informed consent was obtained from the patient for publication of this case report and accompanying images. A copy of the written consent is available for review by the Editor-in-Chief of this journal.

## Competing interests

The authors declare that they have no competing interests.

## Authors' contributions

GM and DO analysed the case, operated the patient, reviewed all patient data and drafted the manuscript. BD carried out the histological analysis wrote the histological part of the paper and contributed to the writing of the final version. IS and KJ were involved in revising the article. All authors reviewed the paper for content and contributed to the writing of the manuscript. All authors approved the final report.
